# Emotion Analysis and Happiness Evaluation for Graduates During Employment

**DOI:** 10.3389/fpsyg.2022.861294

**Published:** 2022-03-24

**Authors:** Lanlv Hang, Tianfeng Zhang, Na Wang

**Affiliations:** ^1^School of Law and Public Affairs, Nanjing Tech University, Nanjing, China; ^2^Sports College, Nanjing Tech University, Nanjing, China; ^3^School of Economics and Management, Nanjing Tech University, Nanjing, China

**Keywords:** emotion analysis, happiness evaluation, restricted Boltzmann machine (RBM), skip connection, deep learning

## Abstract

Happiness can be regarded as an evaluation of life satisfaction. A high level of wellbeing can promote self-fulfillment and build a rational, peaceful, self-esteem, self-confidence, and positive social mentality. Therefore, the analysis of the factors of happiness is of great significance for the continuous improvement of the individual’s sense of security and gain and the realization of the maximization of self-worth. Emotion is not only an important internal factor that affects happiness, but it can also accurately reflect the individual’s happiness. However, most of current happiness evaluation methods based on the emotional analysis belong to shallow learning paradigm, making the deep learning method unexploited for automatically happiness decoding. In this article, we analyzed the emotions of graduates during their employment and studied its influence on personal happiness at work. We proposed deep restricted Boltzmann machine (DRBM) for graduates’ happiness evaluation during employment. Furthermore, to mitigate the information loss when passing through many network layers, we introduced the skip connections to DRBM and proposed a deep residual RBM (DRRBM) for enhancing the valuable information. We further introduced an attention mechanism to DRRBM to focus on the important factors. To verify the effectiveness of the proposed method on the happiness evaluation tasks, we conducted extensive experiments on the statistical data of the China Comprehensive Social Survey (CGSS). Compared with the state-of-the-art methods, our method shows better performance, which proves the practicability and feasibility of our method for happiness evaluation.

## Introduction

Happiness is an emotional experience based on the satisfaction, which also can be seen as an evaluation of life satisfaction. Emotion is a kind of attitude and experience about whether objective things meet their needs, and it is an individual internal factor that affects happiness (Kahneman, 1999). Establishing a mapping relationship between various internal factors of individual emotional performance and happiness can accurately evaluate individual happiness, which has important research significance.

Gamble and Gärling (2012) proposed and tested the conceptualization of happiness. They regarded happiness as a situation-dependent evaluation of hedonic experiences, specifically the valence and activation of current mood, as well as being related to cognitive judgments of life satisfaction. Happiness is a very subjective and complicated concept, the evaluation criteria of which vary from person to person (Lin, 2011). Diener and Lucas (2000) discussed the question of which societal characteristics are likely to enhance subjective wellbeing. Schimmack et al. (2002) investigated the relationship between the cognitive and emotional components of happiness. They concluded that people’s cognitive judgment of life satisfaction depends on people’s emotional experience. Researchers took students as the research group to explore the relationship between students’ social emotional abilities and happiness, and they found that the social emotional abilities of active students usually show a positive relationship with high levels of happiness (Durlak et al., 2011). Studies obtained by Isen (2009) have shown that a better emotional state usually has the following effects on individual performance: enhancing individual creativity, raising individual cognitive flexibility, improving individual problem-solving efficiency, and promoting individual decision-making objectivity.

As a special group, graduates are transitioning from school to society. Their emotional performance and happiness index have an important impact on the development of society. Although graduates have no academic pressure after leaving the school, the social environment they faced is more diverse and complex. They are in an important period of physical and mental self-formation and maturity, facing a series of major issues such as employment, interpersonal relationships, and so on. Especially affected by the coronavirus disease 2019 (COVID-19) epidemic, graduates not only have difficulty for finding a job but also whether they can engage in the current job for a long time or obtain more growth opportunities from the current job is also full of uncertainty. Therefore, current graduates generally have a lower sense of anxiety, insecurity, and happiness. In summary, it is of great social significance to analyze the emotions of graduates during employment and study its influence on personal happiness at work.

College graduates are prone to abnormal emotions such as confusion, depression, and anxiety, which have become increasingly prominent. Studies have shown that the rate of the stress and depression of college students are generally higher all over the world, and their psychology is relatively fragile (Deb et al., 2016). For college students, a series of activities can easily lead to psychological stress and emotional instability, such as settling into university life, learning professional knowledge, interpersonal communication, finding jobs, and pursuing a higher education. Tian (2009) analyzed the emotions of college students in the form of questionnaires. They constructed a complete characteristic system, which indicates that the emotions of college students are closely related to the spiritual, social, and physical levels. To analyze the emotional of college students during the employment period, Lin-ling and San-Qiang (2018) analyzed the emotional tendency of college students who are in the entrepreneurial period by finding the relationship between the emotional and social support of the innovative and entrepreneurial college students. Through the questionnaire survey method, they found that women in the entrepreneurial process are easier to be emotional. Zhao et al. (2016) analyzed the anxiety data of graduates through unconditional Logistic regression. They found that the origin, major, and social work experiences of graduates are the main factors affecting anxiety. In addition, people will feel insecure because of the uncertainty of public health emergencies, leading to various emotional problems. Based on this, Jingting and Yilian (2021) adopted questionnaire surveys and multiple regression methods to analyze the emotional performance of recent graduates during the COVID-19 pandemic. The purpose was to find out whether the main factors of anxiety of recent graduates are related to only child, getting a job, physical condition, etc., and whether the risk factors for recent graduates to produce depression are related to the type of profession, getting a job, and physical condition. Their research results provide a scientific basis for improving the health and happiness index of graduates. Above all, it can be seen that emotion is caused by a series of stimulus interactions, which is the most important factor affecting happiness (Hongyang, 2017).

To explore the emotional factors that affect the happiness of college students, Haobin and Yuanjiang (2012) analyzed the data they collected. The research results show that a high degree of satisfaction with psychological needs is a vital component to improve the happiness of college students, and it is also conducive to dealing with the relationship with themselves, others, group, and society. In addition, it also can deepen the understanding of their own living environment and occupational planning, which is capable of promoting the development of self-worth. Deguang and Xueqin (2019) analyzed the relationship between occupational planning of college students and happiness, which shows that there is a significant correlation between them. In Burr et al. (2011); Anderson et al. (2012), Cheung and Lucas (2015), and Tingting et al. (2016), the research results show that different goals of graduates can bring about different levels of emotional experiences and happiness. In addition, social supports also play an important role in happiness of graduates. Individuals who receive more social supports have more positive emotions and a higher happiness. Moreover, the happiness is also largely affected by socioeconomic status indicators such as income, education, and job. Chengwen (2011) conducted a statistical analysis of the characteristics and *status quo* of the happiness of college students and self-worth and explored the correlation between the both. Senya (2018) adopted the meaning of life as an emotional variable to explore the relationship between the positive emotions of college graduates and their happiness.

In summary, this article takes the features related to people, family, work, life, and society as the main factors affecting emotions and evaluates the happiness of graduates during employment. Traditional emotion analysis and happiness evaluation methods were mainly built on the classic statistical methods. Although being effective, the effectiveness is not satisfactory, resulting in many psychological problems that cannot be detected in time. The traditional data analysis methods belong to shallow machine learning method, which is difficult to deeply explore the essential traits of emotion of graduates during the period of employment, leading to the inaccurate happiness evaluation. To make up for the shortcomings of shallow methods, this article proposes to use deep learning methods to automatically happiness decoding. Therefore, we designed a deep restricted Boltzmann machine (DRBM) to evaluate the happiness of graduates during the period of employment. Furthermore, to mitigate the information loss when passing through many network layers, we introduced the skip connections to DRBM and proposed a deep residual RBM (DRRBM) for enhancing the valuable information. Most importantly, we introduced an attention mechanism to DRRBM to focus on the important factors. Extensive experiments were conducted on the statistical data of the China Comprehensive Social Survey (CGSS). Compared with state-of-the-art methods, our method shows superior classification performance, demonstrating the effectiveness of the proposed method for happiness evaluation.

## Proposed Method

At present, most of the methods of happiness prediction use traditional learning methods, such as K-nearest neighbor (KNN), support vector machine (SVM) (Zhang et al., 2021), and Fuzzy systems (Zhang and Cai, 2020). As mentioned earlier, they belong to the shallow machine learning method, which is difficult to deeply explore the essential traits of emotion of graduates during the period of employment, leading to the inaccurate happiness evaluation. Therefore, this article develops a deep learning-based method, i.e., DRBM, to evaluate the happiness of college graduates during their employment. In the following section, we have introduced the proposed DRBM.

### Restricted Boltzmann Machine

**FIGURE 1:**
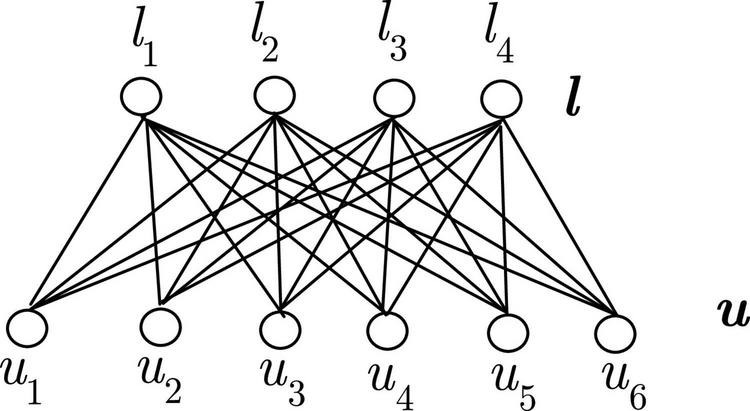
Restricted Boltzmann machine.

Restricted Boltzmann machine (RBM) is a stochastic neural network proposed by Hinton (2007), which is composed of visible and hidden layers. The visible layer takes input data, and the hidden layers reconstruct the input data. The visible layer is directly connected to the first hidden layer. Notably, there are no connections between neurons in the same layer. A simple RBM is schematized in Figure 1. According to Figure 1, the energy of the nodes between the two layers can be expressed as:


(1) $$E\,\left(u,l\right)=-\sum_{i=1}^{m}a_{i}\,u_{i}-\sum_{j=1}^{n}b_{j}\,l_{j}-\sum_{i=1}^{m}\sum_{j=1}^{n}u_{i}\,l_{j}\,w_{i\,j}$$

where *a*_*i*_ and *b*_*j*_ are the biases, *u*_*i*_ and *l*_*j*_ are the binary states of the corresponding nodes, *w*_*ij*_ is the weight between the visible node *i* and the hidden node *j*. Therefore, the probability of the connection between the visible layer and the hidden layer can be formulated as:


(2) $$p\,\left(u,l\right)=\frac{e^{-E\,\left(u,l\right)}}{Z}$$

The log-likelihood of Eq. (2) can be expressed as


(3) $$L\,\left(X_{D}\right)=\sum \log p\,\left(u,l\right)$$

where *X*_*D*_ denotes the training data. According to the theory of contrastive divergence, maximizing the log-likelihood

Equals to minimizing the Kullback-Leibler divergence between the data distribution and the model distribution. Thus, the gradient of the log-likelihood with respect to *w* can be expressed as


(4) $$\frac{\partial \log\left(p\,\left(u,l\right)\right)}{\partial w_{i\,j}}={\left\langle \frac{\partial \log L\,\left(X_{D}\right)}{\partial w_{i\,j}}\right\rangle }_{d\,a\,t\,a}-{\left\langle \frac{\partial \log L\,\left(X_{D}\right)}{\partial w_{i\,j}}\right\rangle }_{m\,o\,d\,e\,l}$$

According to Eqs (1)–(3), Eq. (4) can be simplified as


(5) $$\frac{\partial \log\left(p\,\left(u,l\right)\right)}{\partial w_{i\,j}}={\left\langle u_{i}\,l_{j}\right\rangle }_{d\,a\,t\,a}-{\left\langle u_{i}\,l_{j}\right\rangle }_{m\,o\,d\,e\,l}$$

where ⟨.⟩ represents the expectation of the data or model distribution. Since contrast divergence (CD) can approximate expectations, we used *u*_*i*_*l*_*j*_ to approximate the expectation of ⟨*u*_*i*_*l*_*j*_⟩_*data*_. We used Gibbs sampling of the data-dependent hidden vector to calculate the expectation of ⟨*u*_*i*_*l*_*j*_⟩_*model*_ and express it by ⟨*u*_*i*_*l*_*j*_⟩_*re*_ after further reconstructing the data. Therefore, the change of weight during optimizing the RBM can be expressed as


(6) $$\Delta \,w_{i\,j}=\varepsilon \,\left({\left\langle u_{i}\,l_{j}\right\rangle }_{d\,a\,t\,a}-{\left\langle u_{i}\,l_{j}\right\rangle }_{r\,e}\right)$$

where ε is the learning rate. Therefore, the learning rule for biases can be expressed as


(7) $$\Delta \,a_{i}=\varepsilon \,\left({\left\langle u_{i}\right\rangle }_{d\,a\,t\,a}-{\left\langle u_{i}\right\rangle }_{r\,e}\right)$$

and


(8) $$\Delta \,b_{j}=\varepsilon \,\left({\left\langle l_{j}\right\rangle }_{d\,a\,t\,a}-{\left\langle l_{j}\right\rangle }_{r\,e}\right)$$

### Deep Residual Restricted Boltzmann Machine

**FIGURE 2:**
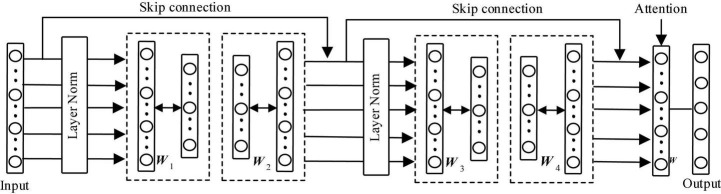
Structure of deep residual restricted Boltzmann machine.

The structure of the proposed deep residual restricted Boltzmann machine (DRRBM) is shown in Figure 2. From Figure 2, we can find that DRRBM is composed of two blocks, two skip connections, and two normalization layers. Each block contains two RBMs and a non-linearity. Given a training data *x* ∈ ℝ^*d*^, where *d* denotes the dimension of *x*. Thus, the feature learning process of DRRBM can be expressed as


(9) $$x_{1}=x+W_{2}\,\sigma \,\left(W_{1}\,L\,a\,y\,e\,r\,N\,o\,r\,m\,\left(x\right)\right)$$

(10) $$x_{2}=x_{1}+W_{4}\,\sigma \,\left(W_{3}\,L\,a\,y\,e\,r\,N\,o\,r\,m\,\left(x_{1}\right)\right)$$

where *W*_1_ ∈ ℝ^*d*×(*d*/2)^ and *W*_2_ ∈ ℝ^(*d*/2)×*d*^ are the weights of RBMs from the first block, *W*_3_ ∈ ℝ^*d*×(*d*/2)^ and *W*_4_ ∈ ℝ^(*d*/2)×*d*^ is the weight of RBMs from the second block. *x*_1_ ∈ ℝ^*d*^ denotes the output of the first block, *x*_2_ ∈ ℝ^*d*^ denotes the output of the second block. σ(.) is an element-wise non-linearity (e.g., RELU). Besides, the layer normalization (Ba et al., 2016) was introduced to the proposed deep model. In addition, to mitigate the information loss when passing through many network layers, we introduced two skip connections to DRBM for enhancing the valuable information. Most of all, to focus on the important features, we further introduced an attention mechanism (Hu et al., 2018) on the output of the second block, as shown in Figure 2.

The output of the second block was then fed into a fully connected layer and a softmax layer.


(11) $$o=x_{2}\,W+b$$

(12) $$p_{i}=\frac{\exp\left(o_{i}\right)}{\sum_{j}^{k}\exp\left(o_{j}\right)}$$

where *W* ∈ ℝ^*d*×*k*^ and *b* are the weight and bias of the fully connected layer. $\sum_{i}^{k}p_{i}=1$, and *k* denotes the number of categories of *x*_2_.

Finally, the prediction of *x* can be represented as:


(13) $$\hat{i}=\arg\,\max_{i}p_{i}$$

## Experiments

In this section, we evaluated the proposed method on the publicly available statistical data of the CGSS.^1^ We first gave the description of the dataset and the data preprocessing. Then, we introduced the adopted comparison methods and their corresponding parameters setting. Finally, we presented and discussed the experimental results.

### Data Preparation and Preprocessing

To verify the effectiveness and the reliability of the proposed model in predicting the happiness of college graduates during the employment period, the CGSS dataset was adopted in this article. This dataset has 139 characteristics, including individual factors (e.g., gender, age, region, and health), family factors (e.g., parents and family income), and social factors (e.g., fairness and public services). Besides, the data can be categorized into 5 classes, in which 1 represents the lowest happiness and 5 represents the highest happiness.

We selected the features, which are mostly related to the employment period of college graduates. First of all, according to the education level, we chose a group of samples related to college graduates for happiness evaluation. Besides, three kinds of features are adopted to analyze the happiness of college graduates during the employment period, namely, individual feature, family feature, and social feature. Individual feature includes three sub-features, namely, basic feature, job feature, and life feature. More specifically, basic feature includes the following factors, including, gender, height, weight, household registration, educational status, and health status; job feature includes employment status, job type, expected job, personal annual income, income satisfaction, and expected salary; life feature includes social entertainment frequency, free time, media usage, socioeconomic status, participate in social security projects, and accommodation conditions. In addition, family feature includes total annual income, economic status, car, real estate, parental education status, and parental employment status. Social feature includes trust factor, fairness factor, and satisfaction factor. The selected features and their descriptions are illustrated in Table 1. In total, we chose 900 training samples and 300 test samples, each of them has 105 features. To avoid the problem of data imbalance, we shuffled the training and test data and randomly selected 900 training data.

**TABLE 1:** Selected features and the descriptions.

Features	Sub-features	Name	Description
Individual feature	Basic information	Gender	Male = 1; female = 2.
		Height	The unit of record is cm.
		Weight	The recording unit is kilograms.
		Household registration	Type and location of household registration.
		Car	Whether there is a car.
		Educational status	Junior college = 10; University degree = 12; Postgraduate and above = 13.
		Health status	Very unhealthy = 1; relatively unhealthy = 2; fair = 3; relatively healthy = 4; very healthy = 5.
	Job information	Employment status	Boss, partners, self-employed, employees, freelance, etc.
		Job type	Full-time work = 1; part-time work = 2.
		Expected job	Boss, partners, self-employed, employees, freelance, etc.
		Personal annual income	The unit of record is RMB.
		Income satisfaction	Describe satisfaction with salary from 1 to 5.
		Expected salary	The unit of record is RMB.
	Life information	Social entertainment frequency	Describe the frequency of social entertainment activities with neighbors and other friends from 1 to 7.
		Free time	Socialize, rest and relax, learn to recharge.
		Media usage	Internet, TV, radio, newspapers, etc.
		Socioeconomic status	Economic status is lower or higher than peers.
		Participate in social security projects	Urban basic medical insurance/new rural cooperative medical insurance/public medical, commercial medical insurance, etc.
		Accommodation conditions	Accommodation area, house ownership
Family feature	-	Total annual income	The unit of record is RMB.
		Economic status	Describe the family’s economic status in the local area from 1 to 5.
		Car	Whether there is a car at home.
		Real estate	Number of Real estates owned.
		Parental education status	Describe different academic qualifications from 1 to 14.
		Parental employment status	Boss, partners, self-employed, employees, freelance, etc.
Social feature	-	Trust	Trust in neighbors, relatives, colleagues, classmates, strangers, etc.
		Fairness	Fairness to the current society.
		Satisfaction	Satisfaction with public education services, medical and health public services, housing security, social management public services, social security, infrastructure, etc.

In the experiment, we preprocessed the data, including filling in missing values, handling outliers, and so on. First of all, we changed the value “–8” (i.e., we can’t answer in the happiness level), to the value “3” (i.e., we don’t know we are happy or not). In addition, the missing values of the features are filled according to different condition, such as the missing value of the number of households was set as 1, the missing value of family income was set as the average of all household incomes, and the others are filled with 0. Finally, the values of features are normalized between zero and one.

### Experimental Setting

To fairly evaluate the performance of the proposed method in predicting the happiness of college graduates during employment, the following six comparison methods are adopted in this article:

K-nearest

(1) neighbor (KNN) (Kramer, 2013);
(2) Support vector machine (SVM) (Noble, 2006);
(3) Back propagation neural network (BPNN) (Lyu and Zhang, 2019);
(4) Deep residual restricted Boltzmann machine (DRRBM);
(5) DRRBM using SVM as classifier (DRSVM) (Tang, 2013);
(6) Attention-based DRSVM (ADRSVM) (Hu et al., 2018).

For above algorithms, we first used the principal component analysis (PCA) (Li et al., 2018) to reduce the dimensionality of the features. KNN and SVM belong to shallow learning methods. For SVM, we adopted one-vs.-rest (OVR) strategy to extend it to the multiclass case. For DRRBM, it has two same blocks and each black composed of four layers. The number of neurons of the four layers are set as 100,50,50, and 100, respectively. Besides, the fully connected layer has 100 neurons. For the algorithm, DRSVM, DRRBM, and SVM are used as its backbone network and classifier. According to the study by Tang (2013), DRSVM can be effectively optimized through end-to-end backward propagation learning strategy. Based on DRSVM, we further introduced an attention mechanism to develop an attention-based DRSVM (called as ADRSVM for simplicity). ADRSVM is capable of automatically identifying the important features for happiness evaluation. In addition, the number of neighbors in KNN is selected from 1 to 5. The trade-off parameters of SVM, DRSVM, and ADRSVM are all obtained by searching the values in set {1e-1, 2e-1, 5e-1, 1e0, 1e1}. For the deep learning methods DRRBM, DRSVM and ADRSVM, the batch size is set as 100. The number of epoch is set as 600, and Adam optimizer is used. The 10-fold cross-validation is used to choose the optimal parameters for all the methods under comparison. In addition, the learning rate is set as 1e-3. To fairly compare the performance of different comparison methods, metrics accuracy (ACC), precision, and F1 on the test data are adopted in this article.

### Experimental Results Analysis

Table 2 shows the experimental results of comparison methods on the test set. According to the experimental results, the following conclusions can be obtained:

**TABLE 2:** Classification results of various comparison algorithms on datasets.

Metrics	Methods					
	KNN	SVM	BPNN	DRRBM	DRSVM	ADRSVM
ACC	77.83%	81.77%	83.66%	86.85%	87.66%	**89.62%**
Precision	80.66%	82.36%	84.83%	88.07%	88.54%	**90.57%**
F1	77.45%	81.68%	83.53%	86.65%	87.28%	**89.76%**

*The best classification results are bold faced.*

(1) Compared with the shallow learning methods KNN and SVM, deep learning methods BPNN, DRRBM, DRSVM, and ADRSVM can achieve better classification performance. As shown in Table 2, the ACC, precision, and F1 of BPNN are 83.66, 84.83, and 83.83%, respectively. Compared with the shallow learning methods SVM and KNN, the absolute ACC, precision, and F1 increase by 1.89, 2.47, and 1.85% and 5.83, 4.17, and 6.08%, respectively. The above results show that deep learning-based methods can achieve better classification results than shallow learning methods. In addition, it can be found that DRSVM outperforms DRRBM, which verifies that SVM can achieve better classification performance than softmax-with-loss function (Tang, 2013).
(2) The ACC, precision, and F1 of the DRRBM are 86.58, 88.07, and 86.65%, respectively. Besides, the ACC, precision, and F1 of the DRSVM o are 87.66, 88.54, and 87.28%, respectively. Overall, DRRBM and DRSVM can achieve better classification performance than BPNN, with a 3.19, 3.24, and 3.12% and 4, 4.16, and 3.75% improvement on the classification metrics ACC, precision and F1. The above results show that the proposed method can indeed mitigate the information loss when passing through many network layers and improve the happiness evaluation performance of college graduates during employment.
(3) In general, ADRSVM achieves better classification results than other comparison methods in all cases. As can be seen from Table 2, the ACC, precision, and F1 of ADRSVM are 89.62, 90.57, and 89.76%, respectively. Compared with BPNN, the absolute ACC, precision, and F1 increased by 5.96, 5.74, and 6.23%, respectively. Compared with DRSVM, the absolute ACC, precision, and F1 increased by 1.96, 2.03, and 2.48%, respectively. The above classification results show that the attention mechanism can automatically choose the valuable information and suppress the misleading information simultaneously, leading better happiness evaluation performance.

## Discussion

To gain a better insight into the performance of the proposed framework, we further considered the impact of different ratios of training data and test data on the performance of the method proposed in this article. After merging the training and test data, we randomly divided them into 4 subsets of equal size. We used the last subset as the test set, and the first one, the first two, and the first three are used as the training set, respectively. In this way, the ratios of the training data and the test data are set as 1:1, 2:1, and 3:1 accordingly. We then compared all methods using different ratios. The classification accuracies on the test data are shown in Table 3. According to the classification results in Table 3, we can obtain the following conclusions. The accuracies of the ADRSVM algorithm in three different ratios are 84.65, 87.95, and 89.62%, respectively. It can be found that the classification performance on the test data increases as the proportion of training data increases. Compared with other algorithms, the ADRSVM algorithm has achieved better classification performance, which verifies the effectiveness of the proposed method in this article. In addition, compared with the DRSVM, ADRSVM achieves an absolute increase in ACC by 2.44, 3.2, and 1.96%. The average ACC of ADRSVM is 87.41%, which achieves an average increase of 2.54% compared with the most competitive method DRSVM. The experimental results demonstrate that the happiness of college graduates during employment has a relatively large relationship with individual features, family features, and social features.

**TABLE 3:** Accuracies of different comparison algorithms with different rations of training and testing data.

Methods	Ratios			
	1:1	2:1	3:1	Avg.
KNN	70.09%	74.50%	77.83%	74.14%
SVM	74.65%	78.86%	81.77%	78.43%
BPNN	76.14%	81.43%	83.66%	80.41%
DRRBM	81.45%	83.02%	86.85%	83.77%
DRSVM	82.21%	84.75%	87.66%	84.87%
ADRSVM	**84.65%**	**87.95%**	**89.62%**	**87.41%**

*The best classification results are bold faced.*

To further verify the effectiveness of the proposed method, we evaluated the statistical significance of the outperformance observed from the experimental results. Pairwise two-tailed *t*-test (Zheng et al., 2018) is adopted to verify whether ADRSVM is significantly superior to the other compared methods on the test data. Table 4 shows the results of the statistical tests. We highlighted the significant results with *p*-value < 0.05. In almost all cases, the null hypothesis that there is no difference in classification performance between ADRSVM and other comparison method is rejected with 95% level, indicating that the outperformance of ADRSVM over the other method is statistically significant. This further verifies the fact that the selected individual features, family features, and social features have a significant impact on happiness during employment.

**TABLE 4:** Significance statistics of different comparison algorithms.

Metrics	ADRSVM vs. KNN	ADRSVM vs. SVM	ADRSVM vs. BPNN	ADRSVM vs. DRRBM	ADRSVM vs. DRSVM
ACC	**0.0037**	**0.0048**	**0.0120**	**0.0315**	**0.0197**
Precision	**0.0079**	**0.0029**	**0.0199**	0.0588	**0.0210**
F1	**0.0010**	**0.0012**	**0.0094**	**0.0046**	**0.0158**

*The best classification results are bold faced.*

According to the above experimental results, we have provided the following suggestions to better improve the happiness evaluation of college graduates during the employment period and promoted the physical and mental health of college graduates.

(1) Colleges should pay attention to the cultivation of students’ employability, which is critical for improving the competitiveness of graduates. Besides, improving the quality of employment of graduates as well as enhancing the matching of labor supply and demand for graduates, so that the skills of graduates can be fully developed. At the same time, colleges should add mental health education courses and set up professional psychological consultation rooms, which are very helpful for students to establish correct values, reduce the incidence of depression, and promote the physical and mental health of students.

(2) The whole society should: (1) strengthen the construction of the humanistic environment; (2) optimize the living environment of graduates; (3) establish a support mechanism; and (4) provide adequate social support for college graduates. Only by doing so, the degree of trust and recognition of college graduates can be increased in the society. College graduates are facing various economic pressures in terms of family and social development. For creating a good policy environment and public service platform, the government should improve public health services, infrastructure construction, housing security, and social security systems, so that the economic burden of college graduates can be reduced. At the same time, to improve the happiness of college students during employment, we should improve governance and service levels to meet the diverse needs of graduates.

## Conclusion

This article proposes a DRBM to evaluate the happiness of college graduates during the employment period. To reduce the loss of information when passing through multiple network layers, we introduced the skip links to DRBM to enhance the valuable information. In addition, to focus on the important factors that are critical to the happiness evaluation, we further exploited the attention mechanism to DRBM. The experimental results on the CGSS dataset verify the effectiveness of the method.

Although the method proposed in this article can significantly improve the performance of graduate happiness prediction, there is still room for improvement. For example, the adopted publicly available CGSS dataset has limited number of college graduates, leaving the proposed method is not verified on the larger dataset. Besides, many other attention mechanisms can be integrated into our proposed framework. In the near future, we will investigate the performance of the proposed method by taking these issues into consideration.

## Data Availability Statement

Publicly available datasets were analyzed in this study. This data can be found here: https://tianchi.aliyun.com/competition/entrance/231702/information.

## Author Contributions

LH was responsible for data processing and data analysis and responsible for manuscript writing. NW was responsible for study design and experimental design. TZ was responsible for manuscript editing. All authors contributed to the article and approved the submitted version.

## Conflict of Interest

The authors declare that the research was conducted in the absence of any commercial or financial relationships that could be construed as a potential conflict of interest.

## Publisher’s Note

All claims expressed in this article are solely those of the authors and do not necessarily represent those of their affiliated organizations, or those of the publisher, the editors and the reviewers. Any product that may be evaluated in this article, or claim that may be made by its manufacturer, is not guaranteed or endorsed by the publisher.

## Footnotes

1
http://cgss.ruc.edu.cn/
